# *CpGislandEVO*: A Database and Genome Browser for Comparative Evolutionary Genomics of CpG Islands

**DOI:** 10.1155/2013/709042

**Published:** 2013-09-25

**Authors:** Guillermo Barturen, Stefanie Geisen, Francisco Dios, E. J. Maarten Hamberg, Michael Hackenberg, José L. Oliver

**Affiliations:** ^1^Departamento de Genética, Facultad de Ciencias, Universidad de Granada, 18071 Granada, Spain; ^2^Laboratório de Bioinformática, Instituto de Biotecnología, Centro de Investigación Biomédica, 18100 Granada, Spain

## Abstract

Hypomethylated, CpG-rich DNA segments (CpG islands, CGIs) are epigenome markers involved in key biological processes. Aberrant methylation is implicated in the appearance of several disorders as cancer, immunodeficiency, or centromere instability. Furthermore, methylation differences at promoter regions between human and chimpanzee strongly associate with genes involved in neurological/psychological disorders and cancers. Therefore, the evolutionary comparative analyses of CGIs can provide insights on the functional role of these epigenome markers in both health and disease. Given the lack of specific tools, we developed *CpGislandEVO*. Briefly, we first compile a database of statistically significant CGIs for the best assembled mammalian genome sequences available to date. Second, by means of a coupled browser front-end, we focus on the CGIs overlapping orthologous genes extracted from *OrthoDB*, thus ensuring the comparison between CGIs located on truly homologous genome segments. This allows comparing the main compositional features between homologous CGIs. Finally, to facilitate nucleotide comparisons, we lifted genome coordinates between assemblies from different species, which enables the analysis of sequence divergence by direct count of nucleotide substitutions and indels occurring between homologous CGIs. The resulting *CpGislandEVO* database, linking together CGIs and single-cytosine DNA methylation data from several mammalian species, is freely available at our website.

## 1. Introduction

Short stretches of CpG dinucleotides (CpG islands or CGIs) predominantly hypomethylated in healthy tissues [1, 2] are key epigenomic markers in mammalian genomes [3]. Almost all housekeeping genes and a half of the tissue-specific genes are associated to CGIs [4]. DNA methylation plays an important role in the origin as well as in the function of CGIs. Aberrant methylation (mostly hypermethylation) of CGIs can lead to several syndromes, such as cancer [5–10]. Moreover, although it has been shown that certain human diseases may have evolutionary epigenetic origins [11, 12], it remains largely unknown how patterns of DNA methylation differ between closely related species and whether such differences contribute to species-specific phenotypes [11]. Some methylation databases [13–15] and CGI databases [16] have been developed, but, to our knowledge, no existing genome browser addresses specifically the evolutionary relationships between the CGIs from different species. To help describing and understanding the function as well as the mechanisms generating and maintaining CGIs within an evolutionary context, we develop here *CpGislandEVO* (http://bioinfo2.ugr.es/CpGislandEVO/index.php). The database, coupled to a powerful genome browser, links together experimental and predicted CGIs, as well as single-cytosine-resolution DNA methylation data from different mammalian species.

Early analyses of CGI evolution were based on compositional comparisons between islands from different species but located on homologous gene contexts [17, 18]. Recently, the rapidly increasing number of sequenced genomes enabled evolutionary studies relying on multiple-sequence alignments [19]. Here, we combine both approaches to envisage accurate sequence comparisons between CGIs located on homologous gene contexts.

## 2. Material and Methods

### 2.1. Genome Assemblies

Updated chromosome sequences for the best assembled mammalian genomes (*Homo sapiens *(hg19),* Pan troglodytes *(panTro3),* Gorilla gorilla *(gorGor3),* Pongo abelii *(ponAbe2),* Macaca mulatta *(rheMac2), *Mus musculus *(mm10), and* Rattus norvegicus *(rn5)) were downloaded from the UCSC genome browser [20].

### 2.2. CGI Predictions

CGIs were predicted by means of an improved version [21] of the *CpGcluster* algorithm [22]. We used the genome intersection as distance threshold to define the clusters of CpG dinucleotides and a *P* value threshold of 1E-5 for the statistical significance. For comparison, the database also includes the CGI predictions for hg19 made by a window-based program [23], as well as the UCSC island track for the different species [20].

### 2.3. Experimental CGIs

Experimental CGI datasets include the 13,277 nonoverlapping promoter regions which are unmethylated in at least one of the two tissues (fibroblast and sperm) analyzed by Weber et al. [24] and the 17,383 CpG-islands experimentally detected in blood cells [25].

### 2.4. Orthologous Gene-Contexts

To ensure that we are comparing truly homologous genome segments, we focus on the CGIs located around orthologous genes extracted from *OrthoDB *[26]. The *OrthoDB* implementation employs a best-reciprocal-hit clustering algorithm based on all-against-all Smith-Waterman [27] protein sequence comparisons. In particular, we take into account all the islands within the gene-body of each of the *OrthoDB* genes. We defined the gene-body as the region extending from 500 bp upstream from the transcription start site (txStart) to 500 bp downstream the transcription stop site (txEnd).

### 2.5. Sequence Comparisons

Base level comparisons of homologous CpG-island sequences from different species were carried out by lifting genome coordinates between assemblies by means of the Galaxy implementation [28, 29] of the *LiftOver* utility, based on the *Chain* and *Net* tracks from the UCSC Genome Browser (http://genome.ucsc.edu/cgi-bin/hgLiftOver/). Default parameters were used, except for the “minimum matching region size for the query” which was set to 8 bp, which corresponds to the smallest CGI length.

### 2.6. Methylation Data

Since the lack of CGI methylation is a very good indicator of function [30], we linked *CpGislandEVO* to a relevant subset of *NGSmethDB* [31, 32], where a wide variety of single-cytosine-resolution methylation methylome maps from different tissues and species are available. Methylomes were obtained with *NGSmethPipe* [33] (http://bioinfo2.ugr.es/NGSmethPipe/) and *MethylExtract* [34] (http://bioinfo2.ugr.es/MethylExtract/), two tools implementing stringent quality controls to minimize important error sources, as for example sequencing errors, bisulfite failures, clonal reads, or single nucleotide variants. Likewise important, the use of a single bioinformatics protocol homogenizes the database content making the different methylomes comparable among each other even if they are from different studies.

### 2.7. Database and Genome-Browser Implementation

The orthologous genes were taken from *OrthoDB* [26], which does not provide information about gene names or coordinates. Therefore, this information is obtained from *ensGene* (using *ensemblIDs* as identifiers) and *refGene* (using gene names) from UCSC [20]. The curated online repository of HGNC-approved gene nomenclature [35] is then used to link names between *ensGene* and *refGene* databases.

We implemented an autocomplete function to help the user locate human genes in *OrthoDB* [26] with at least one orthologous gene in any of the other species (via its gene name or its *UniProtID*). Once chosen a gene name, the *refGene* database (214,898 entries) and, if no results are found, the *ensGene* database (647,600 entries) are searched for this gene. As an output, the chromosome and the start and stop positions of this gene are obtained; a final check for at least one CGI within this gene-body is performed. Then, *OrthoDB* is queried with the *ensemblID* of the human gene, and a table with the *ensemblIDs* of the orthologous genes found in any of the six animal species is generated (Table 1). This table also contains known gene names obtained by converting back *ensemblIDs* via *biomart* databases [36].

When the user introduces a chromosome and an approximate coordinate, the script searches the *ensGene* table for the closest upstream and/or downstream gene with at least one orthologous gene in *orthoDB*, then returning the exact chromosome position and gene name. The corresponding *ensemblID* is then used as above to generate Table 1.

The most recent version (currently 1.9.7) of the cross-platform genome browser *JBrowse* [37, 38] is used to display genes, CpGislands, LiftOver-mapped tracks, and methylation tracks for the hg19 assembly and to compare it to the other six mammalian species. A pair-wise comparison is performed by means of two frames within a window: the top one is always used to display hg19 tracks and the bottom one for one of the six animal species. Note that, by the moment, both frames are not synchronized. This feature will be implemented as soon as Jbrowse_syn is available (http://gmod.org/w/images/a/aa/ISyIPGMODforComparativeGenomics.pdf, slide 15).

Currently, *CpGislandEVO* includes the mammalian genomes with comparable genome-wide methylation data (human, chimpanzee, rhesus monkey, and mouse). In this way, the platform allows the user to compare CGIs from these mammalian species. The number of species and methylation datasets will be increased according to the advent of new comparable genome-wide methylation datasets.

### 2.8. Data Download and Script Availability

The datasets in *CpGislandEVO* can be freely downloaded by the user from NGSmethDB (http://bioinfo2.ugr.es/NGSmethDB/database.php) in a wide variety of standard data formats: BED, GFF3, bedGraph, Wiggle, and so forth. The Perl script for the most recent version of *CpGcluster* is also freely available to download from the group webserver (http://bioinfo2.ugr.es/CpGcluster/CpGcluster.zip).

## 3. Results and Discussion

We first compiled a CGI database (http://bioinfo2.ugr.es/CpGislandEVO/launch.php) for the best assembled mammalian genomes using the *CpGcluster* algorithm [22] with the genome intersection as distance threshold [21, 22, 39]. This setup is especially appropriate for interspecies comparative genomic studies as (i) the distance threshold is directly obtained from the genome sequence and (ii) a *P* value is assigned to each CGI. Therefore, exactly the same criterions are used in all species to detect CpG islands. This is not possible when using algorithms based on sliding windows to predict CGIs, as variations in genome G+C content, O/E ratio, or CpG density cannot be easily taken into account to guarantee an unbiased detection [39]. Second, by means of a specifically designed genome browser based on *JBrowse* [37, 38], we focus on those CGIs located within orthologous gene-contexts [26], thus ensuring that we are comparing CGIs from true homologous sequence segments. Finally, to study sequence divergence at base level between homologous CpG islands, we lifted genome coordinates between assemblies from different species by using the *LiftOver* utility [20].

The *CpGislandEVO* platform first offers the possibility to explore the CGI database obtained with the *CpGcluster* algorithm [21, 22, 39]. After selecting genome assembly and chromosome, the server offers links to (i) *CpGcluster* predictions, (ii) UCSC genome browser [20], and (iii) single-cytosine methylation data by means of a subset (http://bioinfo2.ugr.es/CpGislandEVO/methylation.php) of *NGSmethDB* [31, 32]. Summary statistics for the CGI database and CGI distribution in the orthologous gene bodies of the different species are shown on-line: http://bioinfo2.ugr.es/CpGislandEVO/statistics.php. Second, a coupled genome browser allows sequence comparisons between CGIs located on homologous segments from different species. The user can navigate the database in two ways: (1) by directly introducing a human gene/protein reference name or (2) by providing a chromosome and an approximate coordinate (and then the closest upstream and/or downstream human gene with at least one orthologous gene will be shown). The server first returns the orthologous genes (Table 1) for the query gene with links to *Ensembl* [40] and UCSC [20] genome browsers, as well as to a specific island viewer we have developed on the basis of the *JBrowse* next-generation browser [37, 38]. The *CpGislandEVO* viewer allows the comparative genomics of CGIs in different species.

**Figure 1:**
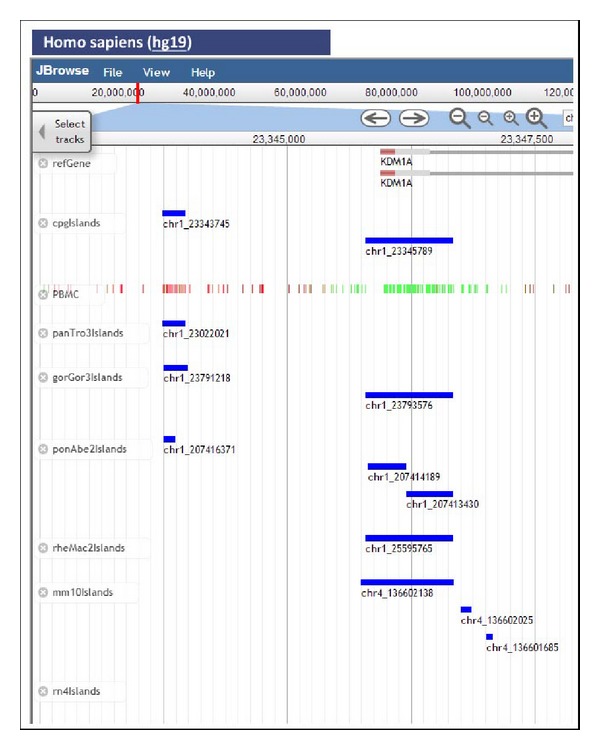
Promoter region of the human gene KDM1A showing CGIs and methylation data for PBMC cells. The lifted homologous CGIs from six other species are shown for comparison. The small methylated human CGI is conserved in three primate species, while the larger unmethylated human CGI is not only conserved in some primates but also in the mouse.

**Figure 2:**
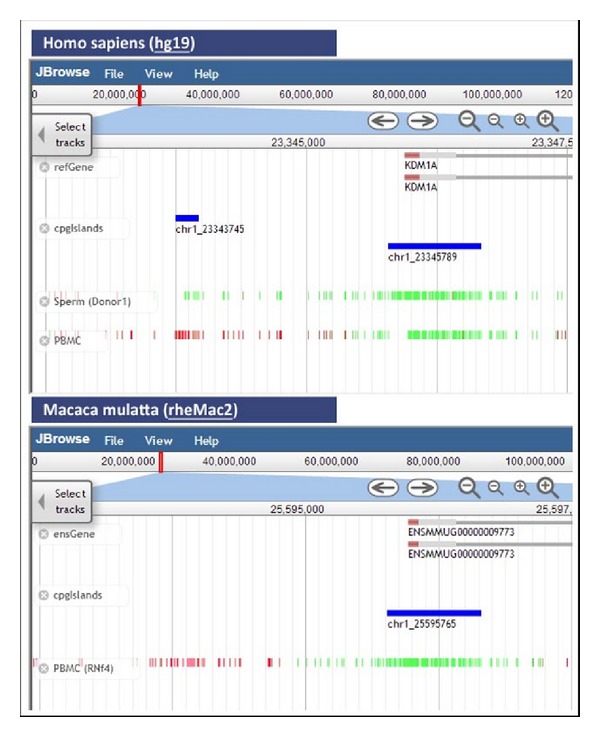
Comparison of the promoter region of the gene KDM1A in human and rhesus monkey using two frames within the same window. The unmethylated CGI is conserved between the two species, while the small human differentially methylated CGI is missing in the rhesus monkey.

As an example, we focus on the human query gene KDM1A for the lysine-specific histone demethylase 1A.  Figure 1 shows the promoter region of this gene and the CGIs and methylation data for PBMC cells. The homologous CGIs from six other species are shown for comparison. The small methylated human CGI is conserved in the three primate species, while the larger unmethylated human CGI is conserved even in the mouse. On the other hand, Figure 2 uses two frames within the same window, to compare the CGIs in the promoter region of the gene KDM1A in human and rhesus monkey. The unmethylated CGI is conserved between the two species, while the small human differentially methylated CGI is missing in the rhesus monkey. In this way, *CpGislandEVO* put together in the same screen information scattered in diverse sources, or only attainable after running different computer programs, thus allowing evolutionary compositional comparisons as well as accurate sequence analyses between islands from different species, but located on homologous gene contexts.

## 4. Conclusions

We have compiled a database of statistically significant CGIs for the best assembled mammalian genomes using an improved version of the *CpGcluster* algorithm [21, 22, 39]. Then, by means of a specifically designed genome-browser based on *JBrowse* [37, 38], we focused on those CGIs located within orthologous gene-contexts [26], thus ensuring that we are comparing CGIs from true homologous genome segments. Finally, by lifting genome coordinates between assemblies from different species, the *CpGislandEVO* platform allows the direct comparison at base level between homologous CpG islands. The evolutionary comparative studies of CGIs can provide insights on their functional role in both health and disease, as well as on the evolutionary mechanisms generating and maintaining these important epigenome markers.

## Figures and Tables

**Table 1a:** (a) Query gene

Species	Gene name	Link Ensembl	Link UCSC	Link JBrowseViewer
*Homo sapiens *	KDM1A	hg_KDM1A_ensembl	hg_KDM1A_ucsc	hg_KDM1A_evoDB

**Table 1b:** (b) Orthologous genes

Species	Gene name (*EnsemblID*)	Link Ensembl	Link UCSC	Link JBrowseViewer
*Gorilla gorilla *	KDM1A (ENSGGOG00000003664)	gorgor_KDM1A_ensembl	gorgor_KDM1A_ucsc	gorgor_KDM1A_evoDB
*Macaca mulatta *	KDM1A (ENSMMUG00000009773)	rhemac_KDM1A_ensembl	rhemac_KDM1A_ucsc	rhemac_KDM1A_evoDB
*Mus musculus *	Kdm1a (ENSMUSG00000036940)	mm_kdm1a_ensembl	mm_kdm1a_ucsc	mm_kdm1a_evoDB
*Pan troglodytes *	KDM1A (ENSPTRG00000000321)	pantro_KDM1A_ensembl	pantro_KDM1A_ucsc	pantro_KDM1A_evoDB
*Pongo abelii *	KDM1A (ENSPPYG00000001747)	ponabe_KDM1A_ensembl	ponabe_KDM1A_ucsc	ponabe_KDM1A_evoDB
*Rattus norvegicus *	Kdm1 (ENSRNOG00000022372)	rn_kdm1a_ensembl	rn_kdm1a_ucsc	rn_kdm1a_evoDB
